# Evolution of glyoxylate cycle enzymes in Metazoa: evidence of multiple horizontal transfer events and pseudogene formation

**DOI:** 10.1186/1745-6150-1-31

**Published:** 2006-10-23

**Authors:** Fyodor A Kondrashov, Eugene V Koonin, Igor G Morgunov, Tatiana V Finogenova, Marie N Kondrashova

**Affiliations:** 1 Section on Ecology, Behavior and Evolution, Division of Biological Sciences, University of California at San Diego, 2218 Muir Biology Building, La Jolla, CA 92093, USA; 2 National Center for Biotechnology Information, National Library of Medicine, National Institutes of Health, Bethesda, MD 20894, USA; 3 Skryabin Institute of Biochemistry and Physiology of Microorganisms, Russian Academy of Sciences, Pushchino, Russian Federation; 4 Institute of Theoretical and Experimental Biophysics, Russian Academy of Sciences, Pushchino, Russian Federation

## Abstract

**Background:**

The glyoxylate cycle is thought to be present in bacteria, protists, plants, fungi, and nematodes, but not in other Metazoa. However, activity of the glyoxylate cycle enzymes, malate synthase (MS) and isocitrate lyase (ICL), in animal tissues has been reported. In order to clarify the status of the MS and ICL genes in animals and get an insight into their evolution, we undertook a comparative-genomic study.

**Results:**

Using sequence similarity searches, we identified MS genes in arthropods, echinoderms, and vertebrates, including platypus and opossum, but not in the numerous sequenced genomes of placental mammals. The regions of the placental mammals' genomes expected to code for malate synthase, as determined by comparison of the gene orders in vertebrate genomes, show clear similarity to the opossum MS sequence but contain stop codons, indicating that the MS gene became a pseudogene in placental mammals. By contrast, the ICL gene is undetectable in animals other than the nematodes that possess a bifunctional, fused ICL-MS gene. Examination of phylogenetic trees of MS and ICL suggests multiple horizontal gene transfer events that probably went in both directions between several bacterial and eukaryotic lineages. The strongest evidence was obtained for the acquisition of the bifunctional ICL-MS gene from an as yet unknown bacterial source with the corresponding operonic organization by the common ancestor of the nematodes.

**Conclusion:**

The distribution of the MS and ICL genes in animals suggests that either they encode alternative enzymes of the glyoxylate cycle that are not orthologous to the known MS and ICL or the animal MS acquired a new function that remains to be characterized. Regardless of the ultimate solution to this conundrum, the genes for the glyoxylate cycle enzymes present a remarkable variety of evolutionary events including unusual horizontal gene transfer from bacteria to animals.

**Reviewers:**

Arcady Mushegian (Stowers Institute for Medical Research), Andrey Osterman (Burnham Institute for Medical Research), Chris Ponting (Oxford University).

## Open peer review

This article was reviewed by Arcady Mushegian (Stowers Institute for Medical Research), Andrei Osterman (Burnham Institute for Medical Research), Chris Ponting (Oxford University). For the full reviews, please go to the Reviewers' comments section.

## Background

**Figure 1:**
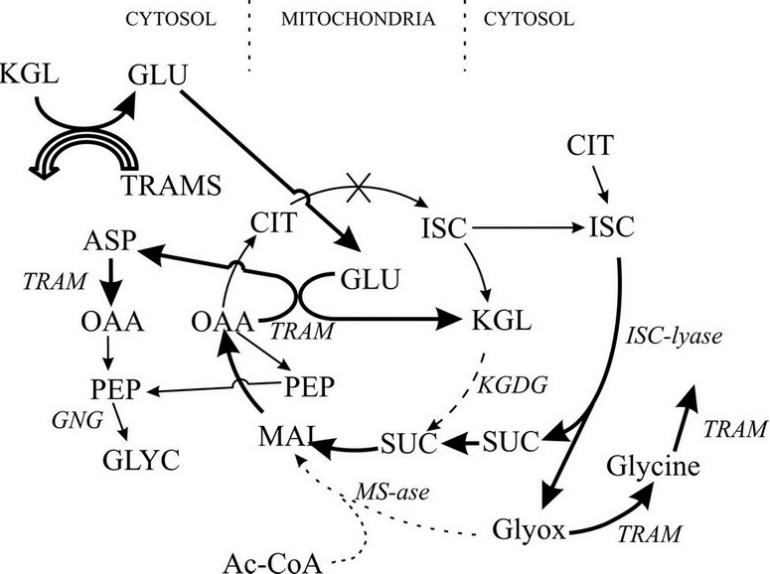
**The complete Krebs, truncated Krebs and glyoxylate cycles**. Abbreviations: α-ketoglytarate -KGL, α-ketoglytarate dehydrogenase – KGDH, acetyl-CoA – Ac-CoA, aspartate – ASP, citrate-CIT, gluconeogenesis – GNG, glutamate – GLU, glycogene – GLYC, glyoxylate-Glyox, isocitrate – ISC, isocitrate lyase – ICL, malate-MAL, malate synthase – MS, oxalacetate – OAA, phosphoenolpyruvate – PEP, succinate-SUC, transaminases (aminotransferases) – TRAM. The truncated Krebs cycle includes the OAA + GLU -> ASP + KGL reaction that is catalyzed by glutamate-glyoxylate-aminotransferase [58, 59]. TRAM reactions in mitochondria and cytosol are connected by common amino and keto acids. Thick lines represent rapid reaction steps, dashed lines – slow and easily inhibited steps, crossed out like is the blocked aconitase reaction, dotted like – malate synthase pathway that may have been recently lost in placental mammal common ancestor.

**Figure 2:**
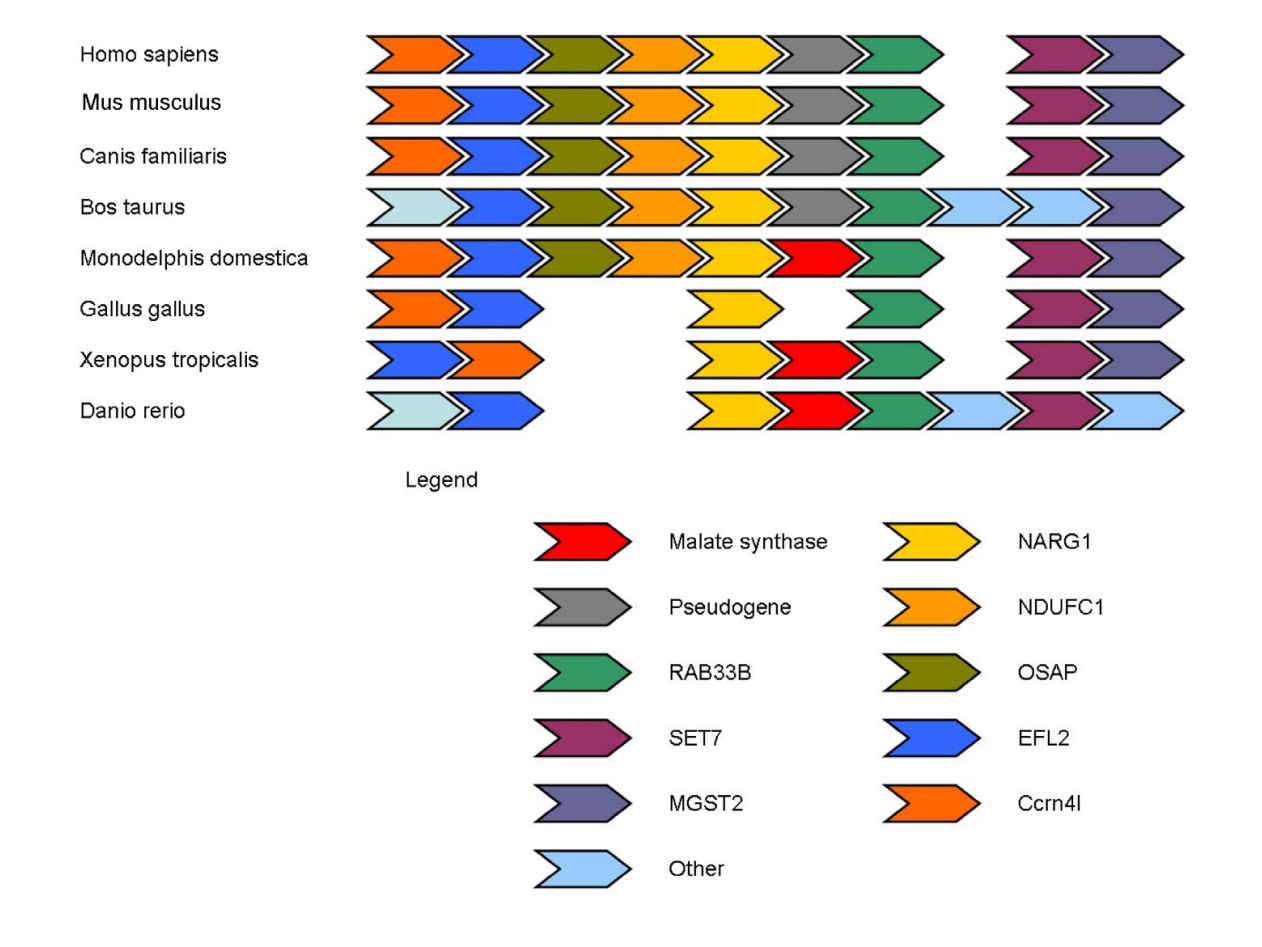
The syntenic region around malate synthase orthologs in completely sequenced Coelomate genomes.

Glyoxylate cycle is a distinct, anaplerotic variant of the tricarboxylic acid (TCA) cycle the net effect of which is the conversion of two molecules of acetyl-CoA to succinate gluconeogenesis. The glyoxylate cycle shares three of the five involved enzymes with the TCA cycle. skips the two rate-limiting decarboxylation steps of the latter, which are catalyzed by isocitrate dehydrogenase and α-ketoglutarate dehydrogenase (Figure 1; [1,2]). The glyoxylate cycle deviates from the TCA cycle when isocitrate, instead of being decarboxylated into α-ketoglutarate by isocitrate dehydrogenase, is converted into glyoxylate and succinate by isocitrate lyase (ICL). Malate synthase (MS) completes the shortcut by producing malate from glyoxylate and acetyl-CoA (Figure 1). Succinate produced by the glyoxylate cycle is utilized, primarily, for carbohydrate synthesis. Both ICL and MS are essential for the function of this pathway and are thought to be dedicated, glyoxylate cycle-specific enzymes such that their activities are often considered to be signatures of this pathway [2].

It is widely accepted that the glyoxylate cycle operates in bacteria [2], fungi [3], some protists [4,5], and plants [6]; in addition, recent reports [7,8] identified a bifunctional enzyme in nematodes with both ICL and MS activities that apparently evolved by the fusion of the respective genes (see also [2]). Although several authors reported ICL and/or MS activity in other Metazoa, including birds [9], reptiles [10,11], and placental mammals [12-20], the claim that the glyoxylate cycle functions in animals other than nematodes remains controversial [21,22]. One of the major problems with regard to the existence of the glyoxylate cycle in Metazoa is the failure to identify the ICL and MS genes in metazoan genomes, except for those of the nematodes. Here, we undertake a bioinformatic analysis aimed at detection of orthologous genes for the two glyoxylate cycle-specific enzymes in the available complete and draft genomes of various animals and reveal the existence of a MS pseudogene in placental mammals. We further examine the phylogenies of these enzymes and derive evolutionary scenarios that include multiple horizontal gene transfer (HGT) events.

## Results

### Isocitrate lyase and malate synthase genes and pseudogenes in animals

In addition to the previously identified ICL homologs in the nematodes *Caenorhabditis *and *Strongyloides*, a putative ICL gene has been annotated in the mosquito *Anopheles gambiae *and the sea anemone *Nematostella vectensis*, and we also found an incomplete homolog in the mosquito *Aedes aegypti*. However, the extremely high similarity of the protein sequence of the mosquito and bacterial genes (only 20% divergence between *A. gambiae *and *E. coli*) and the lack of introns in the mosquito sequence strongly suggested a bacterial contamination. Indeed, such contamination appears to be common at least in the *A. gambiae *genome (S. L. Mekhedov and EVK, unpublished observations). In contrast, the predicted sea anemone ICL sequence contained introns and was identical to several EST sequences. In addition, we identified ICL homolgos among EST sequences for two other Cnidarians (*Acropora millepora*, *Hydractinia echinata*) and several nematodes (*Ancylostoma ceylanicum*, *Globodera rostochiensis*, *Heterodera glycines*, *Parastrongyloides trichosuri*, *Pristionchus pacificus*, *Meloidogyne hapla*, *Meloidogyne javanica*, *Xiphinema index*). The high sequence conservation of ICL (Table 1) implies that, if intact copies of this gene were present in other completely sequenced metazoan genomes, we would have been able to detect them easily. Thus, it appears that, of all Metazoa with sequenced genomes, only nematodes and Cnidaria encode ICL.

**Table 1:** Protein divergence (p-distance) of isocitrate lyase from selected genomes.

	*Euglena gracilis*	*Arabidopsis thaliana*	*Saccharomyces cerevisiae*	*Caenorhabditis elegans*	*Dictyostelium discoideum*	*Chlamydomonas reinhardtii*	*Escherichia coli*	*Brucella melitensis*
*Arabidopsis. thaliana*	0.743							
*Saccharomyces cerevisiae*	0.758	0.479						
*Caenorhabditis elegans*	0.714	0.606	0.612					
*Dictyostelium discoideum*	0.708	0.600	0.608	0.369				
*Chlamydomonas reinhardtii*	0.716	0.601	0.614	0.380	0.357			
*Escherichi coli*	0.703	0.580	0.606	0.403	0.383	0.360		
*Brucella melitensis*	0.710	0.607	0.613	0.275	0.344	0.348	0.363	
*Sulfolobus solfataricus*	0.717	0.566	0.598	0.397	0.394	0.370	0.379	0.369

In contrast, apparent MS orthologs are readily identifiable in several animal genomes including nematodes (*C. elegans*, *C. briggsae*, *C. remanei*), cnidarians (*N. vectensis*), insects (*A. gambiae*, *A. aegypti*, *Bombyx mori*), echinoderms (*Strongylocentrotus purpuratus*), and vertebrates (*Danio rerio*, *Tetraodon nigroviridis*, *Fugu rubripes, Xenopus tropicalis*, *Monodelphis domestica*). The *Ornithorhynchus anatinus *(platypus) and *Oryzias latipes *(fish) genomes also appear to possess the MS gene. In addition, for an insect (*Spodoptera frugiperda*), two cnidarians (*Hydractinia echinata, Acropora millepora*), a variety of nematodes (*Ancylostoma caninum*, *Ancylostoma ceylanicum*, *Globodera rostochiensis*, *Heterorhabditis bacteriophora*, *Heterodera glycines*, *Heterodera schachtii*, *Meloidogyne arenaria*, *Meloidogyne incognita*, *Meloidogyne javanica*, *Parastrongyloides trichosuri*, *Pristionchus pacificus*, *Trichostrongylus vitrinus*, *Trichuris vulpis*, *Xiphinema index*), a primitive chordate (*Branchiostoma floridae*, lancelet), and several vertebrates (*Trichosurus vulpecula*, *Hippoglossus hippoglossus*, *Oryzias latipes*, *Salmo salar*, *Pimephales promelas*, *Xenopus laevis*, *Gasterosteus aculeatus*, *Fundulus heteroclitus*), we detected at least one EST corresponding to the MS gene. However, these sequences were excluded from further analysis because they did not cover the entire coding sequence. None of the detected MS homologs from animals have been characterized experimentally although some of them are annotated in Genbank as proteins similar to the nematode malate synthase/isocitrate lyase bifunctional protein. In the genome sequence of the sea anemone (a Cnidarian) *N. vectensis*, we detected two distinct MS genes; however, we strongly suspect that one of these is a contamination from an animal source because ESTs corresponding to this genes were not detectable, and it clustered with vertebrates and sea urchin in a phylogenetic tree that included insect MS genes as an outgroup (data not shown).

Despite the lack of experimental evidence, there are several indications that animal MS homologs (in addition to those from nematodes) are functional enzymes. Firstly, the coding sequences of these genes do not contain nonsense or frameshift mutations or large insertions or deletions, and the protein sequences retain the conserved motifs characteristic of bacterial MS (data not shown). Secondly, the gene structure is preserved between closely related species, and all introns have the canonical splicing sites (GT...AG), suggesting that the transcripts of these genes are properly spliced. Thirdly, most of these sequences contain regions that are identical or nearly identical to EST sequences. Finally, the rate of non-synonymous substitutions in these genes is substantially lower than the rate of synonymous substitutions, which indicates that these genes are subject to purifying selection at the level of the protein function (Table 2).

**Table 2:** Pairwise comparisons of malate synthase genes in Coelomata genomes.

	*Anopheles gambiae*	*Aedes aegypti*	*Bombyx mori*	*Strongylocentrotus purpuratus*	*Danio rerio*	*Takifugu rubripes*	*Tetraodon nigroviridis*	*Xenopus tropicalis*	*Monodelphis domestica*
*Anopheles gambiae*		65.2561	66.8261	66.3264	65.3852	21.7031	59.0776	62.9706	65.6821
Aedes aegypti	0.1874		64.6634	66.2250	66.4475	63.7981	65.8231	12.0822	66.8287
*Bombyx mori*	0.4153	0.4429		64.8519	7.2214	66.0626	66.5992	66.4243	65.9305
*Strongylocentrotus purpuratus*	0.4654	0.4957	0.5145		7.0848	27.4334	18.0603	56.6819	64.4132
*Danio rerio*	0.4748	0.4742	0.5009	0.3354		3.6424	6.9404	12.1555	12.9374
*Takifugu rubripes*	0.4522	0.4805	0.4817	0.3317	0.1844		0.3413	12.8377	37.2786
*Tetraodon nigroviridis*	0.4414	0.4585	0.4728	0.3298	0.1919	0.0441		36.8792	19.8208
*Xenopus tropicalis*	0.4877	0.5024	0.4543	0.3374	0.2440	0.2583	0.2530		3.5119
*Monodelphis domestica*	0.4395	0.4348	0.4682	0.3013	0.2222	0.2211	0.2189	0.1533	

The lower half of the table shows the rates of evolution in nonsynonymous sites, and the upper half shows the rates of evolution in synonymous sites. Most of the synonymous evolution rates were at the saturation levels. However, in each case, the estimated nonsynonymous substitution rate was significantly lower than the corresponding synonymous rate, which is indicative of purifying selection at the amino acid sequence level.

We have not found the MS gene in more than 15 sequenced genomes of placental mammals. Given the substantial number of genomes searched, the high conservation of the MS sequence in other animals (Table 2), and the fact that the closely related opossum sequence was used as the query to search other mammalian genomes, it seems most unlikely that we have missed this gene. A TBLASTN search of the placental mammal genome sequences using the opossum MS sequence as the query showed several marginally significant hits to the same genomic region where the MS is found in the opossum genome. However, these searches detected only a small portion of the MS sequence in placental mammal genomes, and some of the identifiable sequences contained stop codons (Table 3). A comparison of the gene orders in the corresponding genomic regions shows that the sequences similar to MS were located in the exact position occupied by the MS gene in other animals (Figure 2). Thus, it appears that these searches detect the true orthologs of MS but the gene was inactivated and became a pseudogene in the placental mammal lineage. Although the general synteny conservation in this genomic region extends to the chicken genome (Figure 2), we found no evidence of a functional gene or a pseudogene in that region in chicken. Thus, it appears likely that the MS gene has been independently disrupted beyond recognition in the chicken genome. Similarly, the gene order is conserved between *A. gambiae *and the numerous sequenced genomes of *Drosophila *species (data not shown), however, we have not been able to find any traces of a pseudogene in *Drosophila*.

**Table 3:** Malate synthase pseudogenes in placental mammals

Species	Number of exons found by TBLASTN	Percent of opossum MS gene covered by TBLASTN hits	Number of stop codons in hits
*Bos Taurus*	2	8.6%	0
*Canis familiaris*	2	10.6%	0
*Cavia porcellus*	Not Found		
*Dasypus novemcinctus*	3	26.5%	3
*Echinops telfairi*	2	21.4%	3
*Felis catus*	4	32.5%	0
*Homo sapiens*	2	27.0%	0
*Loxodonta africana*	2	14.8%	1
*Macaca mulatta*	4	18.2%	0
*Mus musculus*	Not Found		
*Oryctolagus cuniculus*	1	13.5%	2
*Rattus norvegicus*	Not Found		
*Sorex araneus*	2	17.5%	1
*Myotis lucifugus*	2	18.0%	1
*Otolemur garnettii*	2	22.2%	1
*Spermophilus tridecemlineatus*	2	25.2%	1

The presence of the detectable MS pseudogene in several mammalian genomes seemed unexpected because pseudogenes are usually not recognizable after ~100 million years that separate the mammalian orders from their common ancestor, as indicated by several studies of human and mouse genome divergence [23,24]. However, the rodent lineage appears to evolve substantially faster than other mammalian orders [25-27], and indeed, the MS pseudogene was not detected by genome-wide TBLASTN searches of rodent genomes (Table 3). Thus, some ancestral pseudogenes might have evolved beyond recognition only in the fastest evolving mammalian orders but remain recognizable in others.

### Horizontal gene transfer in the evolution of glyoxylate cycle enzymes in eukaryotes

**Figure 3:**
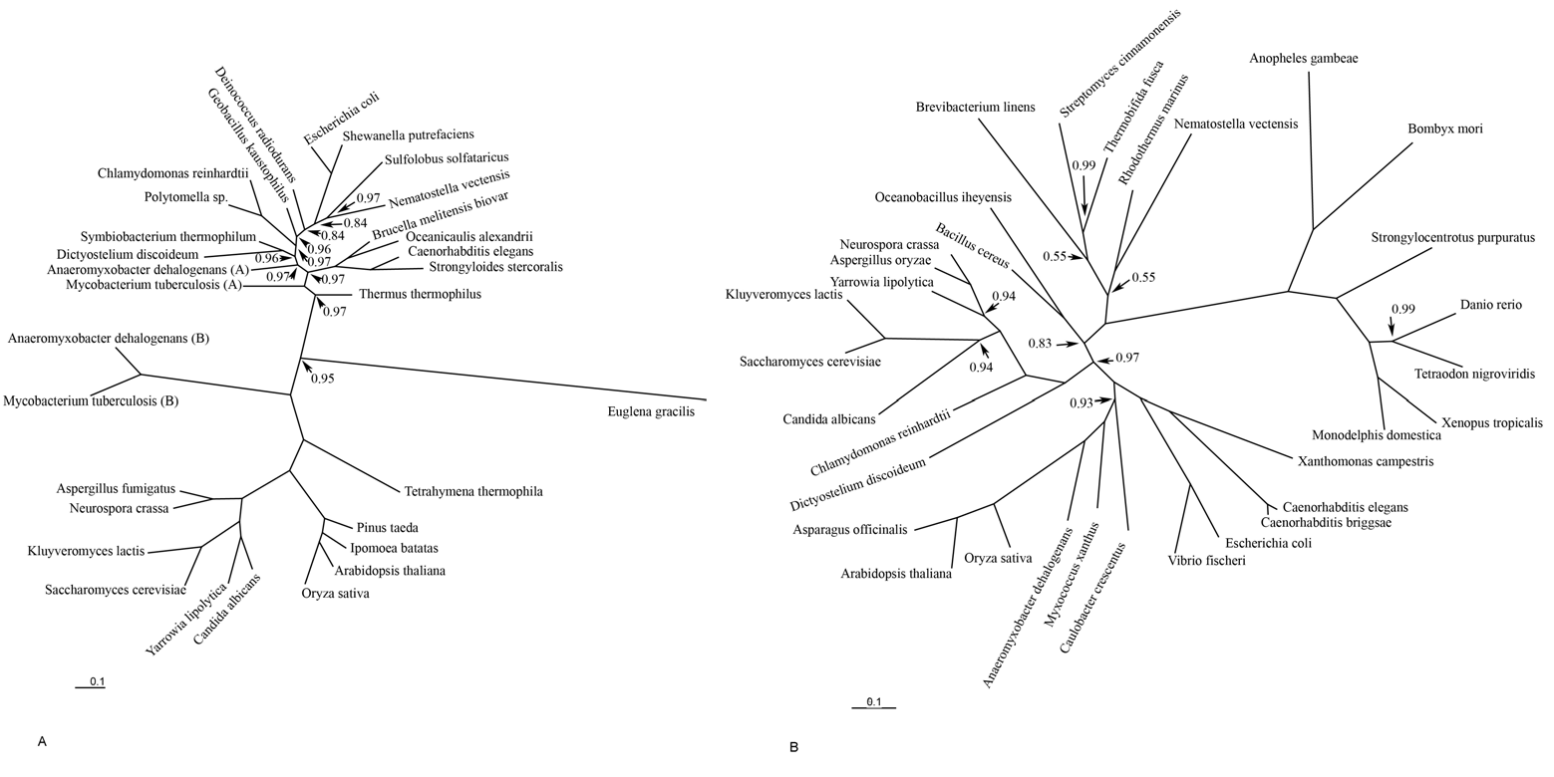
**The phylogenies of isocitrate lyase (a) and malate synthase (b)**. The tree was constructed using the Bayesian approach with the posterior probabilities shown on the tree. Posterior probabilities of 1.0 are not shown.

Several lines of evidence suggest that there was extensive HGT of bacterial MS and ICL genes into several eukaryotic lineages [28]. The two genes are fused to form a bifunctional gene in the nematodes and *Euglena*, but in the nematodes, the ICL domain precedes the MS domain, whereas *Euglena *has the reversed domain order [5]. Since ICL and MS are encoded in the same *ace *operon in many bacteria, and the gene order in the operon also varies, it has been suggested that nematodes and *Euglena *acquired these genes via HGT from bacteria with the respective gene orders in the *ace *operon [5]. This hypothesis predicts that, in phylogenetic trees, the domains from the bifunctional eukaryotic genes should cluster with homologs from bacteria that have the same gene order in the *ace *operon. In practice, testing this prediction was not a straightforward task. The ICL domain of the bifunctional enzymes of the nematodes showed very high (>70% identity) sequence similarity to the ICL of α-proteobacteria, in particular, those of the genus *Brucella*, and clustered with these bacterial proteins in the phylogenetic tree (Figure 3). However, in the sequenced α-proteobacterial genomes, the MS gene is located in a region distant from the ICL gene such that there is no *ace *operon. Interestingly, the MS domain sequence of the bifunctional nematode enzyme showed by far greater similarity to the MS from a different assemblage of bacteria, in particular, several species of Gram-positive bacteria of the genus *Bacillus *(~57% identity), in contrast to the much lower similarity to the MS of *Brucella *(~25% identity). In the phylogenetic tree of MS, the nematode sequences did not cluster with any specific bacterial clade but rather was positioned at the root of the bacterial subtree (Figure 3). This might result from acceleration of evolution of the MS domain in the nematode lineage and/or the absence of the actual bacterial source of the nematode gene in the current databases. Taken together, the evidence seems to be compelling for the horizontal transfer of the ICL-MS gene from bacteria into the nematode lineage. The most likely scenario would involve HGT into the nematode lineage of a "hybrid" *ace *operon containing a "proteobacterial-type" ICL and a "Gram-positive-type" MS; a bacterial genome with such a "hybrid" *ace *operon (or the actual fusion of the two genes) remains to be discovered.

Interestingly, the ICL and MS genes in the cnidarian genome appear to be encoded in tandem but on the opposite strands, in the convergent, 5'-5' orientation. However, without further sequencing of this genomic region from other cnidarians, it is unclear if the two genes originate from an ancestral *ace *operon but one of them was inverted in the sea anemone or the current gene organization is an assembly artifact. In the reconstructed phylogenies (Figure 3), the sea anemone ICL and MS genes cluster within different sets of bacteria which, as in the case of nematodes, might reflect acquisition of a "hybrid" ace operon from an unknown bacterial source.

**Figure 4:**
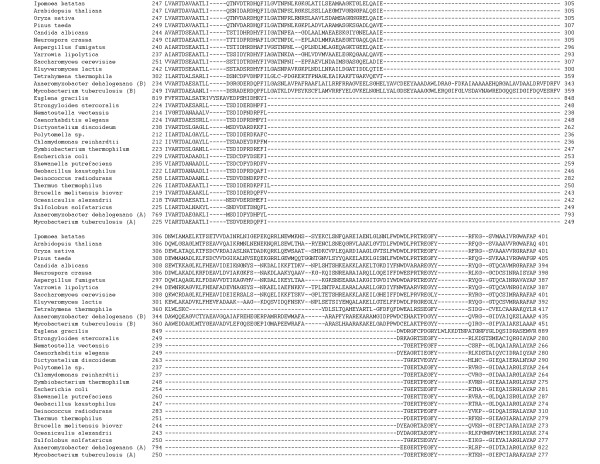
Multiple alignment of isocitrate lyase in the vicinity of the plant- and fungal-specific insertion.

**Figure 5:**
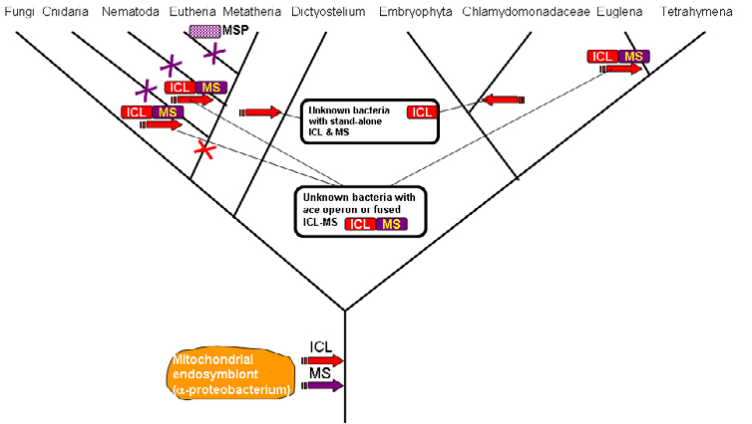
**The inferred scenario for ICL and MS during eukaryotic evolution**. The schematic shows only selected branches of the phylogenetic tree of eukaryotes, those that are relevant to inferred events in the evolution of the glyoxylate cycle. Block arrows show horizontal gene transfer, and crosses show gene loss; MSP stands for malate synthase pseudogene.

With regard to the bifunctional gene of *Euglena*, specific phylogenetic inferences were not feasible because of the extremely high rate of evolution in the *Euglena *lineage (Figure 3). Nevertheless, acquisition of a MS-ICL operon via HGT remains a distinct possibility. In addition to the apparent HGT of the bifunctional gene into the nematode lineage, the phylogenetic tree of ICL suggests at least three other independent instances of bacteria-to-eukaryotes HGT – into the *Nematostella*,*Dictyostelium *and Chlamydomonadaceaelineages (Figure 3a). The rest of the eukaryotic ICLs, i.e., those from plants, fungi, and the ciliate *Tetrahymena*, form a well-defined clade with one of the two copies of the ICL gene from *Mycobacteria *and *Anaeromyxobacter dehalogenans *(Figure 3a). The monophyly of this clade is additionally supported by the presence of a distinctive inserted domain which seems to be a derived shared character (Figure 4). Thus, considering all the evidence, the most likely evolutionary scenario for ICL seems to include the following events (Figure 5): i) early acquisition of the ICL gene by an ancestral eukaryote from bacteria, most likely, the mitochondrial endosymbiont, ii) evolution of the insertion domain, possibly, by internal duplication with subsequent radical divergence, iii) secondary, reverse HGT of the ICL gene from an early eukaryote to a bacterium (possibly, an ancestral *Mycobacterium*), iii) loss of the ICL gene at the outset of animal evolution, iv) at least five additional HGTs from bacteria to eukaryotes, resulting in displacement of the ancestral eukaryotic form of ICL by various bacterial forms in chlamydomonads, *Dictyostelium*, *Euglena*, cnidaria, and nematodes. In the case of nematodes and Euglena, and possibly, cnidaria as well, HGT was accompanied by fusion of ICL and MS genes, probably, facilitated by the juxtaposition of these genes in the respective bacterial *ace *operons (it is also conceivable that the fusion occurred within a bacterial genome prior to the HGT). An alternative scenario would involve the origin of the eukaryotic-type ICL in a distinct bacterial lineage (possibly, an ancestral *Mycobacterium*) with subsequent HGT into an early prokaryote. Given that all bacteria that have the eukaryotic-type ICL also possess a second, typical bacterial ICL, this scenario seems less likely. Regardless of the exact evolutionary scenario of ICL, the unusual, for animals, acquisition of the bifunctional ICL-MS enzyme by nematodes via HGT from a bacterial source appears undeniable.

**Figure 6:**
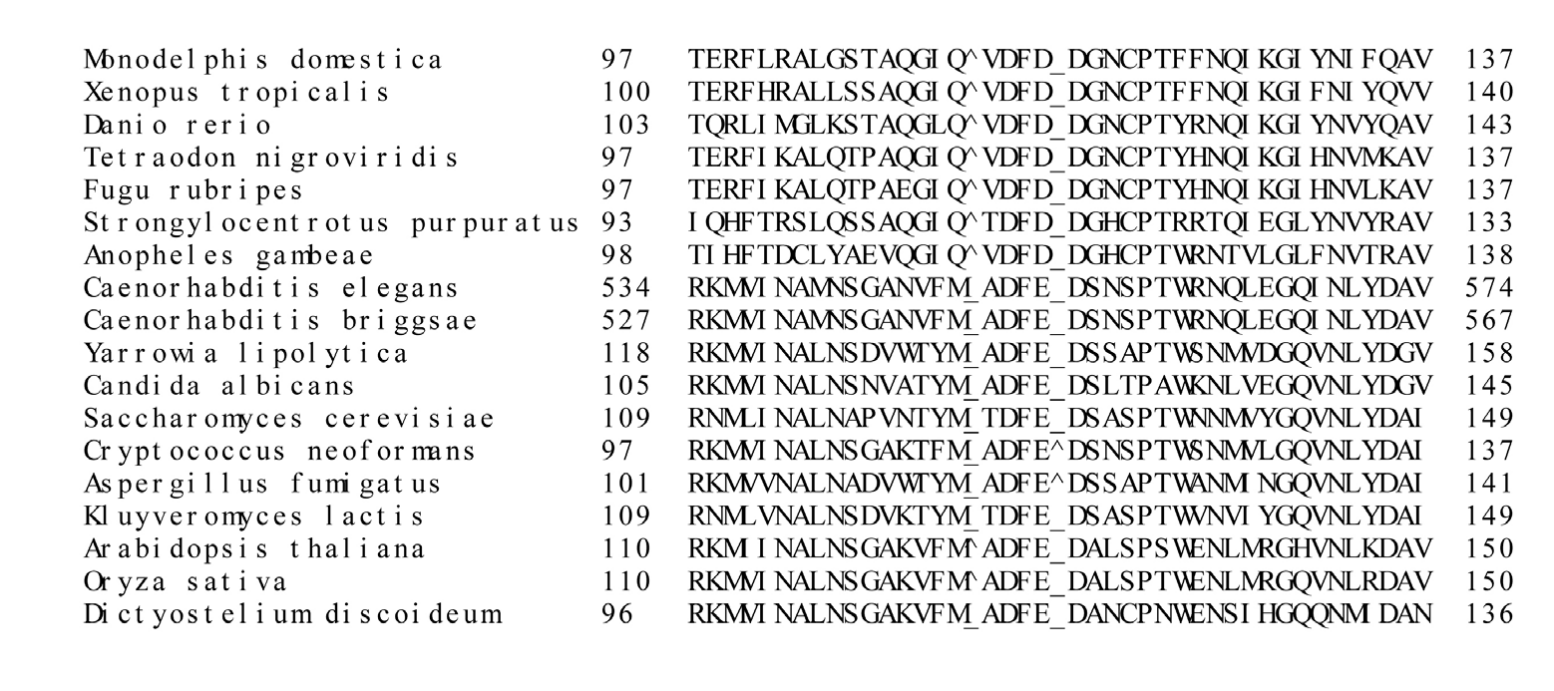
**Multiple alignment of malate synthase in the vicinity of the intron common to higher animals and plants**. The presence of an intron is shown with the carrot symbol (^) while the absence of one is shown with an underscore (_).

The MS phylogenetic tree is less well-resolved than the ICL tree (Figure 3b), and the multiple alignment of MS has not revealed any plausible derived shared characters, such as lineage-specific large inserts (Figure 4), complicating the inference of the evolutionary scenario. In order to assess the monophyly of eukaryotic MS, we compared intron positions in eukaryotic genes. Many introns are conserved in orthologous genes from plants and animals, whereas independent gain of introns in the same position in different lineages is unlikely [29,30]. Therefore, the presence of even one shared intron strongly suggests monophyly of the respective genes as opposed to origin via independent HGT events. Indeed, although plants and coelomate animals did not form a clade in the MS tree (Figure 3b) and instead appeared to cluster with different bacterial species, plant and coelomate MS genes shared one intron in the same position (Figure 6), which is best compatible with their origin from a common eukaryotic ancestor. In contrast, the nematode and the cnidarian gene do not share introns with other animal, fungi or plant genes (or with each other), in either the MS and ICL sequences, which is consistent with a history of HGT (see above).

## Discussion and conclusion

The evolution of glyoxylate cycle enzymes, ICL and MS, seems to have involved a remarkable array of events. These include at least three independent gene fusions, in nematodes, *Euglena*, and *Anaeromyxobacter dehalogenans*, multiple HGTs, and gene loss, in particular, in animals. The probable acquisition of the bifunctional ICL-MS gene via HGT from bacteria in nematodes and cnidarians is of special note because the very reality of acquisition of new genes by animals via HGT from bacteria is a highly controversial topic, and there are very few strongly supported cases [31-35]. The HGT of the bifunctional ICL-MS is supported by multiple lines of evidence, namely: i) unusually high similarity between the respective animal and bacterial genes, at least, in the case of ICL, ii) confident placement of the ICL and MS domains of the animal bifunctional enzymes within specific bacterial branches in phylogenetic trees, iii) juxtaposition of ICL and MS that is not seen in other eukaryotes but is common in bacteria (*ace *operons), iv) absence of shared intron positions between the bifunctional enzymes and the stand-alone homologs from other eukaryotes. Collectively, these observations seem to make the nematode and cnidarian ICL-MS true "smoking guns" of HGT from bacteria to specific lineages of animals. We believe that this is an important proof of principle that justifies a systematic search for other such cases.

Given that most archaea lack the glyoxylate cycle enzymes (with a few exceptions that, in all likelihood, can be attributed to HGT from bacteria ([36], Figure 3)), it appears most likely that eukaryotes originally acquired these genes from the mitochondrial endosymbiont. The ICL gene was lost early in metazoan evolution but was reacquired in the nematode and cnidarian lineages. In contrast, the MS gene was generally retained throughout the evolution of the eukaryotes but became a pseudogene in placental mammals. Combined with conflicting experimental data, these observations stress the conundrum around the function of the glyoxylate cycle-specific enzymes in coelomate animals. One possibility is that these enzymes, ICL and MS, have been lost in Coelomates, but MS was recruited to perform a new function. However, there is currently no experimental evidence of any function of MS other than its involvement in the glyoxylate cycle, and no indication of acceleration of evolution of the MS gene in the Coelomate lineage, which would be expected in the case of a substantial change of function. Alternatively, the ICL gene might have been lost after a different, perhaps, distantly related or unrelated gene evolved the isocitrate lyase function in the Coelomate lineage – a potential case of non-orthologous gene displacement, a fairly common evolutionary phenomenon [37]. This explanation is compatible with several experimental reports that demonstrate the presence of the ICL and MS activities in Coelomates [9-20]. However, since the validity of these experimental results has been challenged [21,22], determination of the function(s) of the MS in Coelomates would be a major step towards the resolution of the conundrum.

The functional significance of the pseudogenization of MS in placental mammals and possible independent loss of MS in birds is another enigma. One possibility is that the generally higher transaminase activity in warm-blooded mammals [38] enhanced the removal of the toxic glyoxylate through transamination by several glyoxylate-animotransferases ([39-43], Figure 1), rendering the MS activity non-essential. The alternatives are that, even if other Coelomates possess the glyoxylate cycle, placental mammals and birds have lost it entirely, or yet another gene evolved the malate synthase function in an additional case of non-orthologous gene displacement.

The extreme evolutionary mobility of the glyoxylate cycle enzymes might seem puzzling although, as far as prokaryotic metabolic pathways are concerned, it is not entirely unprecedented [44]. The key biological consideration appears to be that the two enzymes of the glyoxylate cycle comprise a compact, readily transferable functional unit, especially, when the two genes are juxtaposed or fused. Acquisition of this unit immediately endows the recipient with new metabolic capabilities – to produce succinate and to eliminate the toxic glyoxylate – which could be a selective advantage, at least, under some metabolic regimes.

## Methods

We employed a series of similarity searches for isocitrate lyases and malate synthases in GenBank, and complete and draft genomes of all Metazoans available at NCBI and EMBL. The genomes searched and the parameters of the searches where identical for the two proteins.

The *Saccharomyces cerevisiae *sequences (isocitrate lyase – NC_001137; malate synthase – NC_001146) to the non-redundant protein sequence database at NCBI [45] using the BLASTP program [46] in order to identify all Metazoan homologues that have already been annotated in protein sequence. This approach identified isotrate lyase genes that were annotated in *A. gambiae *(XP_561347), *C. elegans *(NP_503306), *C. briggsae *(CAE62276), *Strongyloides ratti *(BAD89436) and *S. stercoralis *(AAF00535), and malate synthase genes in *A. gambiae *(XP_315354), *C. elegans *(NP_503306), *C. briggsae *(CAE62276), *S. purpuratus *(XP_782946), *D. rerio *(XP_685378) and *T. nigroviridis *(CAF91513). These homologues were identified unambiguously, with low expectation values (E < 1 × 10^-30^) and with at least 40% identity.

All genes that were predicted from complete genomes rather than obtained by direct sequencing of mRNAs (genes from *A. gambiae, D. rerio *and *T. nigroviridis *in this case) were checked for consistency of the annotation. To do this, the predicted protein sequence were mapped to the complete genomes available at the UC Santa Cruz Genome Browser [47] using the BLAT program [48] and checked for correct splice sites in introns (GT...AG), for start and stop codons in the first and last exons, and for the absence of nonsense or frameshift mutations in the retrieved sequence. Where appropriate, the annotation was modified to fit these criteria, and the resulting protein sequence checked by alignment to closest homologues that have been sequenced directly from an mRNA.

The next step of the sequence query was a recursive BLAST search of the available draft and complete Metazoan genomes. First, all isocitrate lyase and malate synthase protein sequences, which were identified in the step described above, were compared with the nucleotide sequences of these genomes using the TBLASTN program [46]. When a homolog was found in one of the genomes, it was annotated according to the sequence similarity with the respective protein sequence, and then checked for correct splice sites, start and stop codons, and the lack of frameshift and nonsense mutations. To complete the search cycle, the newly identified genes was then used as a query in a new TBLASTN search of the Metazoan genomes. The following genomes were queried: *Homo sapiens*, *Pan troglodytes*, *Macaca mulatta*, *Mus musculus*, *Rattus norvegicus*, *Cavia porcellus*, *Canis familiaris*, *Felis catus*, *Bos taurus*, *Dasypus novemcinctus*, *Echinops telfairi*, *Loxodonta africana*, *Oryctolagus cuniculus*, *Sorex araneus*, *Myotis lucifugus*, *Otolemur garnettii*, *Spermophilus tridecemlineatus*, *Monodelphis domestica*, *Ornithorhynchus anatinus*, *Gallus gallus*, *Xenopus tropicalis*, *Takifugu rubripes*, *Tetraodon negroviridis*, *Danio rerio*, *Ciona savignyi*, *Ciona intestinalis*, *Strongylocentrotus purpuratus*, *Bombyx mori*, *Aedes aegypti*, *Anopheles gambiae*, *Tribolium castaneum*, *Nematostella vectensis, Caenorhabditis elegans, Cenorhabditis briggsae *and twelve *Drosophila *species. To marginalize the possibility that the ICL and MS gene sequences are the result of bacterial contamination, we checked for the presence of an introns by BLAT [48] and ESTs by a TBLASTN search [46] in dbEST [49].

Finally, position-specific search implemented in PSI-BLAST [50] was used to search for possible missed homologues among the annotated genes from human, mouse, rat and *Drosophila *genomes and a ScanProsite [51] search of all genes in the UniProt (Swiss-Prot and TrEMBL) [52] and PDB [53] databases. This procedure has not revealed any Metazoan isocitrate lyase or malate synthase sequences that were not picked up with the BLASTP or TBLASTN searches.

Syntenic regions of genomes revealed by BLAST and BLAT searches of genes adjacent to the ICL and MS genes; only assembled genomes were considered. Multiple protein alignments were constructed using the MUSCLE program [54] with default parameters and manually checked for errors and for consistency of the alignment with the ScanProsite [51] ICL and MS amino acid patterns. Rates of synonymous and nonsynonymous evolution were calculated with the PAML package [55].

Phylogenetic trees were constructed by two methods, the neighbor joining procedure with 10,000 bootstrap replicates using with the MEGA program [56] and the Bayesian inference approach implemented in the MrBayes program [57] run with a GTR model assuming a gamma-distribution of substitution rates across sites for 1 million iterations (mcmc ngen = 1000000 in MrBayes). The two methods revealed, largely, congruent phylogenies.

## Abbreviations

α-ketoglutarate -KGL, α-ketoglutarate dehydrogenase – KGDH, acetyl-CoA – Ac-CoA, aspartate – ASP, citrate-CIT, gluconeogenesis – GNG, glutamate – GLU, glycogene – GLYC, glyoxylate-Glyox, isocitrate – ISC, isocitrate lyase – ICL, malate-MAL, malate synthase – MS, oxalacetate – OAA, phosphoenolpyruvate – PEP, succinate-SUC, transaminases (aminotransferases) – TRAM.

## Reviewers' comments

### Reviewer's report 1

Arcady Mushegian, Stowers Institute for Medical Research (with additional contribution from Manisha Goel).

This is an interesting work, starting to trace the unusual path of evolution of malate synthase and isocitrate lyase in animal kingdom, with additional discussion of what might have been going on with these genes in bacteria.

I suggest that the authors do the following:

1. Due diligence with the databases of unfinished genomes: I did a quick tblastn against the environmental sequence genomes at NCBI and saw at least one entry that codes for the same domain tandem as the two-domain nematode protein: is it one ORF or two, from a nematode or perhaps from a bacterium? The unfinished bacterial genomes – perhaps the donor of two genes to the nematode lineage can be identified among them?

**Author response: ***We significantly expanded the scope of searches in the revised version. Indeed, there are some very similar sequences of the ICL-MS fused nematode gene in the environmental sequence database. We find sequences that are highly similar to the nematode gene in two different configurations, with ICL and MS or not fused. Unfortunately, however, it is impossible to tell whether these sequences are from bacteria or eukaryotes, and therefore, we cannot use this information to resolve any of the issues regarding the potential donor of the ICL-MS fused gene in the nematodes*.

When the authors say 'gene transfer into the nematode lineage', how do they know it is not an earlier event (search Schmidtea genome traces perhaps, also Coelenterata)? The same databases, plus ESTs, are needed to account for additional ICLs (I think I can see some in corals).

**Author response: ***Since the submission of the first draft of this manuscript, the cnidarian Nematostella vectensis genome draft has been completed, and now we have included the ICL and MS gene sequences found in this genome into our analysis. Interestingly, the sea anemone genes appear to cluster with bacterial genes as well, albeit with different lineages of bacteria than the nematode genes. We believe that HGT of the fused gene (or operon, with subsequent fusion) in the nematode lineage is the most parsimonious solution. An earlier HGT, e.g., to the common ancestor of Metazoa, would require genes losses in addition*.

### Reviewer's report 2

Andrei Osterman, Burnham Institute for Medical Research

The strength of the manuscript by F. Kondrashov et al. on Evolution of glyoxylate cycle enzymes in Metazoa is in the detailed analysis of possible evolutionary scenarios that included multiple horizontal gene transfer (HGT) events from bacteria to eukaryotes beyond a symbiotic ancestor of extant mitochondria. Such a case-study is a best possible contribution to the heated debates on this exciting albeit highly controversial topic. Based on a solid comparative analysis of genomic sequences of multiple bacterial and eukaryotic species, which included the delineation of intron/exon structures and "pseudogenized" regions in genomes of Metazoa, the authors presented several plausible scenarios. While differing in details, all of them inevitably include several independent cross-kingdom HGT and gene fusion events. In that regard this paper is a highly recommended reading and thinking material. A weaker aspect of this study is a relatively low impact on our understanding of a metabolic driving force behind these amazing events. Despite a heroic attempt to build on the existing fragmental and highly controversial biochemical data, an emerging picture remains largely obscure. The above notion hardly argues against the authors of this study, but rather provides another illustration of a profound disregard of the basic metabolic biochemistry by the overwhelming majority of the experimental research community in the post-genomic era. A juxtaposition of a monumental effort (and quite a stunning progress) on elucidating minute details of signaling cascades, transcription machinery and other complex systems versus an apparent lack of any drive to finally straighten out basic questions such as: (i) presence or absence of malate synthase activity or (ii) actual function of a malate synthase homologs in placental animals, can hardly be reconciled other than by a popular misconception of the actual depth of our knowledge of basic metabolism. Contrasting this problem and putting it in a fundamental evolutionary context is another (likely unintended) impact of this article.

Overall, I firmly support the publication of the submitted article in "Biology Direct", and I believe that it is a perfect fit for the mission of this distinguished Journal.

**Author response: ***Actually, the original motivation behind this article was to apply computational approaches in an attempt to resolve the paradox of the glyoxylate cycle in mammals and birds: several laboratories have reported that this pathway was functional but the participant enzymes could not be identified. As it happens, the paradox only deepened as we ascertained the presence of "orphan" MS in many animals and pseudogenes in mammals. So it was very much our intent, indeed, our primary goal, to attract attention to the mysterious function of the animal MS, and hopefully, to stimulate relevant biochemical experimentation. The discovery of interesting cases of HGT was, in a sense, a by-product of our research, even if it might have the greatest general impact of the observations reported here*.

### Reviewer's report 3

Chris Ponting, Oxford University

This is an interesting study aiming at resolving the long-standing issue of whether malate synthase and isocitrate lyase genes are functional in many animal genomes. It is argued that the malate synthase gene is functional in non-eutherian mammals, other vertebrates, echinoderms and arthropods, but that eutherians have lost this gene through pseudogenization. Meanwhile, the isocitrate lyase gene appears to be absent from all animals with the notable exception of nematode worms (where it is fused with malate synthase). It is argued that these two genes were transferred horizontally into several eukaryotic lineages.

One of the issues with which the authors had to contend was of contamination. I agree that the homologues purported to be in mosquito genome sequences appear to result instead from contamination from bacterial sources. Certainly there is evidence that the mosquito genomes are contaminated with these bacterial genes, particularly because they appear to be single exon genes present between clone gaps in unplaced sequence. Contamination might also be an explanation for the postulated horizontal gene transfer of ICL into *Dictyostelium *and *Chlamydomonas *lineages, but this wasn't (but should have been) considered by the authors.

**Author response:***Contamination can be a serious problem in studies such as this one. We have done our best to exclude the possibility of bacterial or other contaminations of Metazoan sequences by examining gene structure, EST sequences, and level of sequence similarity. We concluded that the ICL sequence from the mosquito Anopheles gambeae and one of the MS sequences from the cnidarian Nematostella vectensis are likely contaminants. By contrast, other Metazoan ICL and MS genes, including those of Dictyostelium and Chlamydomonas, contained introns, and most have several independent EST sequences in GenBank which effectively rules out bacterial contamination*.

A main finding of this report is that the isocitrate lyase gene is absent from "completely sequenced metazoan genomes". Whilst this appears true, there are numerous isocitrate lyase ESTs from cnidarians apparent in public databases. The authors will need to determine whether these represent independent horizontal or else vertical acquisitions, or else consider whether they are contaminants of the type seen in mosquito genomes.

**Author response:***Indeed, these EST sequences are identical to the ICL and one of the MS genes from the genome. Thus, we have included the sequences from the recently completed genome draft of Nematostella vectensis; however, we have not included the EST sequences of other species with ESTs for which we could not obtain the cognate genomic sequences*.

Table 2 shows PAML non-synonymous and synonymous substitution rates between diverse metazoans. The vast majority of these estimates are not meaningful since saturation of substitution will have occurred, and so Table 2 should not be kept. If the authors believe me to be in error here, they should demonstrate their point using classic tests for saturation.

**Author response: ***Indeed many of the values reported in that table are beyond saturation. However, what is clear from this table is that the nonsynonymous divergence (which is nowhere near saturation levels) is much lower than the rate of synonymous divergence, thus demonstrating functional constraint. Therefore, we opted to keep the table after adding a note of caution to the reader regarding the interpretation of the estimates of the synonymous divergence*.

I do not think the authors have presented sufficient evidence that there "was extensive HGT of bacterial MS and ICL genes into several eukaryotic lineages". They imply that these genes have been acquired independently from separate bacterial sources before being fused in nematodes. They discount the possibility that the evolutionary rate of nematode malate synthase might be particularly high without explanation and instead choose a scenario that the bacterial source is as yet unknown. I do not consider that this provides "compelling" evidence for horizontal transfer of both ICL and MS genes from bacteria, which has implications for the title of the manuscript.

**Author response: ***Suppose the genes in the nematode, as well as Chlamydomonas, Dictyostelium and Nematostella (but not other eukaryotic species) have experienced a substantial acceleration of the evolution rate. In order for such acceleration to result in the nesting of these sequences within bacterial clades (which is what we observe) this acceleration must have been coupled with extensive ****convergent ****evolution as well. We believe that such convergent mode of evolution is highly unlikely, to say the least, and a much more parsimonious explanation for the phylogenetic tree reported here is a series of HGT events. Thus, we stand by our statement that the evidence for several cases of HGT from bacteria to specific eukaryotic lineages, including nematodes and Cnidaria, is compelling, and there was no reason to modify the title of the paper. Partly in consideration of these comments, the discussion of HGT was expanded in the revised version of the paper*.

Moreover, if one considers that the horizontal acquisition of ICL by slime mold and *Chlamydomonas *lineages might instead be accounted for by contamination of sequences by bacterial sources, there is even less evidence for the "four additional HGTs from bacteria to eukaryotes" proposed.

**Author response: ***As discussed above in our response to Mushegian's comments, it is highly unlikely that the source of ICL and MS sequences from Chlamydomonas, Dictyostelium and Nematostella is bacterial contamination since the genes in these species have introns and ESTs corresponding to these genes are available for all three o fthese species. We mention these observations in the revised text*.

## Competing interests

The author(s) declare that they have no competing interests.

## Authors' contributions

FAK, EVK, IGM, MNK designed the study, FAK and EVK carried out the bioinformatic analysis, and all authors participated in drafting of the manuscript, and approved the final version.
